# Violent Sleep-Related Behaviours in a Patient With Post Traumatic Stress Disorder (PTSD) and Traumatic Brain Injury (TBI) : A Case of Suspected Medication-Induced Rapid Eye Movement (REM) Sleep Behaviour Disorder

**DOI:** 10.1192/bjo.2026.11866

**Published:** 2026-06-30

**Authors:** Jessie Muchoki

**Affiliations:** Western Health and Social Care Trust, Derry, United Kingdom

## Abstract

**Aims:**

REM sleep behaviour disorder (RBD) is characterised by loss of physiological REM atonia, resulting in dream-enactment behaviours that may be violent and pose significant risk to patients and bed partners. RBD may be idiopathic, secondary to neurological disease, or medication-induced. Medications, including antidepressants and sedative-hypnotics, have been implicated in precipitating or unmasking RBD, particularly in individuals with underlying neuropsychiatric vulnerability and structural brain injury.

**Methods:**

A 60-year-old man who was diagnosed with PTSD following a severe physical assault 30 years ago. He also sustained a scalp laceration, severe bruising to his body, hearing impairment, memory problems, and anxiety problems. Documentation of prior neurological and neuroimaging follow-up was unavailable.

He presented with a six-year history of progressively worsening sleep-related behaviours. These included snoring, talking, shouting, odd sounds, nightmares, increased restlessness, abnormal nocturnal movements, falls from bed when startled, and a recent episode of violent behaviour during sleep resulting in injury to his partner. He had a longstanding psychotropic polypharmacy, including paroxetine, carbamazepine, risperidone, and zopiclone. Collateral history highlighted worsening impairment of his memory and concentration levels, and recent changes in his personality.

Physical examination revealed an abnormal gait, positive Romberg’s test and tremors of his upper limb, with involuntary tic-like movements and repetitive blinking. Cognitive assessment revealed significant deficits in memory, recall, attention and language. Due to a childhood history of petite mal epilepsy, an electroencephalogram was done and it showed no epileptiform discharges. Neuroimaging identified chronic gliotic change and encephalomalacia in the left frontal lobe, consistent with previous injury. Previous oximetry and multi-channel sleep studies excluded sleep apnoea.

Zopiclone and paroxetine were gradually discontinued, and sertraline and melatonin were initiated. Risk management involved separate sleeping arrangements. Subsequently, violent sleep-related behaviours resolved. The patient was referred to Memory Services for further neurocognitive assessment.

**Results:**

The combination of violent dream-enactment behaviours, associated injury to a bed partner, absence of epileptiform activity, structural frontal lobe injury and improvement following gradual withdrawal of long-term paroxetine and zopiclone supported a diagnosis of likely medication-induced RBD in the context of TBI.

**Conclusion:**

This case illustrates the risks of diagnostic overshadowing in patients with established psychiatric diagnoses and highlights the importance of considering organic and iatrogenic causes when new behavioural disturbance emerges, such as violent sleep-related behaviours. This case highlights the importance of awareness of the long-term neuropsychiatric sequelae of TBI and regular medication review and rationalisation to minimise iatrogenic harm

